# Changes in Socio-Economic Inequality in Neonatal Mortality in Iran Between 1995-2000 and 2005-2010: An Oaxaca Decomposition Analysis

**DOI:** 10.15171/ijhpm.2016.127

**Published:** 2016-09-24

**Authors:** Mostafa Amini Rarani, Arash Rashidian, Ardeshir Khosravi, Mohammad Arab, Ezatollah Abbasian, Esmaeil Khedmati Morasae

**Affiliations:** ^1^Department of Health Management and Economics, School of Public Health, Tehran University of Medical Sciences, Tehran, Iran.; ^2^Health Management and Economics Research Center, Isfahan University of Medical Sciences, Isfahan, Iran.; ^3^Deputy of Public Health, Ministry of Health and Medical Education, Tehran, Iran.; ^4^Department of Economics, Bu-Ali Sina University, Hamadan, Iran.; ^5^Department of Public Health, Qom University of Medical Sciences, Qom, Iran.; ^6^Centre for System Studies (CSS), Hull University Business School (HUBS), Hull York Medical School (HYMS), University of Hull, Hull, UK.

**Keywords:** Neonatal Mortality, Socio-Economic Inequality, Oaxaca Decomposition, Iran

## Abstract

**Background:** Exploring changes in health inequality and its determinants over time is of policy interest. Accordingly, this study aimed to decompose inequality in neonatal mortality into its contributing factors and then explore changes from 1995-2000 to 2005-2010 in Iran.

**Methods:** Required data were drawn from two Iran’s demographic and health survey (DHS) conducted in 2000 and 2010. Normalized concentration index (CI) was used to measure the magnitude of inequality in neonatal mortality. The contribution of various determinants to inequality was estimated by decomposing concentration indices in 1995-2000 and 2005-2010. Finally, changes in inequality were investigated using Oaxaca-type decomposition technique.

**Results:** Pro-rich inequality in neonatal mortality was declined by 16%, ie, the normalized CI dropped from -0.1490 in 1995-2000 to -0.1254 in 2005-2010. The largest contribution to inequality was attributable to mother’s education (32%) and household’s economic status (49%) in 1995-2000 and 2005-2010, respectively. Changes in mother’s educational level (121%), use of skilled birth attendants (79%), mother’s age at the delivery time (25-34 years old) (54%) and using modern contraceptive (29%) were mainly accountable for the decrease in inequality in neonatal mortality.

**Conclusion:** Policy actions on improving households’ economic status and maternal education, especially in rural areas, may have led to the reduction in neonatal mortality inequality in Iran.

## Background

Global neonatal mortality rate (NMR) has declined from 33 deaths/1000 live births in 1990 to 20 in 2013.^1^ However, despite substantial progress in decreasing average neonatal mortality, neonatal mortality is still unequally distributed across different socio-economic groups within and across societies.^2-9^ Although national average levels are critical, merely focusing on national averages could be misleading,^8^ as such progress could be achieved by improving the health of the wealthy while overlooking the health of the poor.

Measuring socio-economic inequality in neonatal survival and understanding the gap in neonatal death between the well-off and the worst-off people provides valuable evidence for health policy-makers indicating whether neonatal healthcare programs have managed to narrow the inequality or not. For policy purposes, in addition to measuring socio-economic inequality, we need to answer at least two questions. First, what are the determinants of inequality at a particular point in time? Second, how socio-economic inequality and its contributors have changed over time? The former could be revealed through applying socio-economic inequality decomposition approach and the latter by using Oaxaca-type decomposition technique.

Decomposition of concentration index (CI) is now widely used to determine contribution of factors to socio-economic inequality.^10-16^ Quantifying contributions of determinants to health inequality yield reliable information and links monitoring of health inequality to the knowledge of its determinants.^17^ Therefore, use of decomposition findings by policy-makers may lead to better measures to tackle socio-economic inequality in health (the “gap approach”) instead of actions to tackle average health problems (“the level approach”).^18^ Thus, not only decomposition of socio-economic inequality in neonatal mortality would indicate more specifically the type of policies and places that facilities should be directed towards to address leading causes of inequality, but it also would unearth factors beyond the scope of health sector which require intersectoral collaboration in order to bear substantial reduction in inequality between the poor and the better-off.

One of the approaches to explaining changes in socio-economic inequality over time, proposed by Wagstaff et al,^15^ is Oaxaca decomposition method. This method allows one to decompose changes in CI into changes in determinants of health and also changes in elasticities of those determinants.^19^

From 1990 to 2015, Iran has developed and implemented five national economic, social, and cultural development plans (NDPs). All of these five-year NDPs tried to challenge economic inequalities by prioritizing rural areas and low-income groups.^20^ Development of primary healthcare networks and medical services, improvement of family planning, the establishment of universal health insurance scheme, and the increase of healthcare coverage in rural areas and among underserved population have been of some NDPs induced measures. Enactment of these national development programs alongside some well-targeted child health programs such as integrated management child illness (IMCI), well baby care program, and a surveillance system for 1-59 months child mortality may have resulted in successful neonatal mortality reduction in Iran over last decades. According to child mortality report 2011, NMR (deaths per 1000 live births), has declined from 28 to 14 since 1990 to 2010 in Iran.^21^

Despite such a considerable reduction in the average level of neonatal mortality, the Gini coefficient has been somehow steadily increasing over the years mounting to 0.409 in 2010.^22^ Furthermore, inequality in healthcare expenditure favoring the rich has also increased over the last decades in Iran.^20^ These trends raise the above-mentioned questions of how such increasing socio-economic inequalities have affected neonatal mortality distribution, and how determinants of socio-economic inequality in neonatal mortality have changed over time in Iran. Therefore, the present study, using decomposition approach and Oaxaca method, aimed to answer these two questions.

## Methods

### Data

The data used in this study were taken from Iran’s demographic and health survey (DHS) conducted in 2000,^23^ and Iranian multiple indicators demographic and health survey (IrMIDHS) carried out in 2010.^24^ The sampling design of DHS 2000 was stratified single stage (equal size) cluster sampling with unequal sampling probabilities. The sample population included 2000 urban households and 2000 rural households in each 28 provinces of the country plus 2000 households in Tehran. In overall, 113 957 households were selected into that survey. The sampling design in IrMIDHS 2010 was stratified multi-stage (equal size) cluster sampling in which minimum sample size was estimated to be 400 households in each province. The overall sample comprised of 31 300 households.^25^ The survey in 2000 was conducted based on the DHS program which contained following questionnaires: household-related questionnaire, women’s questionnaire, and questionnaire for under-five children. The survey in 2010 was done based on the United Nations Children’s Fund (UNICEF) multiple indicator cluster survey (MICS). In addition to three above-mentioned questionnaires, MICS contained a specific questionnaire for under-five children in three southeastern provinces in Iran (including questions about malaria) and also a standard anthropometry tool for under-five children. Data from women’s and children’s questionnaires that had same variables in the DHS 2000 and IrMIDHS 2010 were used in the present study. However, the household-related questionnaire had some differences in two surveys, ie, besides the list of asset variables used in 2000; some newer asset variables were added into 2010 study. Consequently, the authors examined the inconsistencies in questions and response options across two surveys and cleaned the data by deleting duplicates and omitting neonates’ observations with incorrect birth and death age registries. Moreover, the data sets were validated by comparing our neonatal mortality estimates with those of other national surveys for similar time periods. As estimates of other national surveys were relatively close to our estimates, we concluded that our data sets were valid. To make datasets comparable and to get the estimates right, three features of sampling design, cluster sampling and stratification (unequal selection probabilities) that arise from design and data collection procedure in surveys were considered.^26^ Ignorance of these factors often leads to incorrect estimates and overestimation of standard errors.

### Variables Definition

Neonatal death was selected as a binary outcome variable, ie, whether each of the live-born neonates (≤28 days) of the women interviewed was still alive or not. Due to the relative scarcity of neonatal mortality, one-year death estimates were not adequately precise,^5^ and could not ensure enough births to reduce effects of sampling error.^27,28^ Hence, survival status of neonates was investigated during a 5-year observation period before the surveys in 2000 and 2010. Accordingly, 45 646 live births from 1995-2000 covered by DHS 2000 and 10 604 live births from 2005-2010 covered by MIDHS 2010 were investigated.

Independent variables were selected based on a conceptual framework proposed by Mosley and Chen,^29^ including household’s economic status, mother’s educational level, mother’s age at delivery, neonate gender, place of residence (urban or rural), the mother’s history of abortion/stillbirth, risky birth interval (under 24 months), skilled prenatal care, skilled birth attendants, use of modern contraceptive, and possession of a hygienic toilet.

Household economic status was constructed by principal component analysis (PCA),^19,30^ using available data from 113 215 households in DHS 2000 and 30 870 households in MIDHS 2010. In the absence of direct data on income and expenditure in DHS, one popular and widely used approach for assessing the household’s economic status is to apply PCA to construct a wealth index from information on household ownership of durable assets and housing characteristics.^31^ The use of economic status measure constructed from the combination of durable assets and housing characteristics, informs us of living standards and access to public services that cannot be adequately recognized by direct measures (such as income or expenditure). Thus, when we discuss rich/poor-favoring inequality, it is an issue of inequality in household’s conditions and consumptions rather than only their income or expenditure.

In this study, the following asset variables were used in PCA model in 2000: number of rooms per capita, access to piped drinking water, use of natural gas for heating and cooking, access to a heating system, and possession of bathroom, refrigerator, television, telephone, car, motorcycle, and bicycle. The assets used in the PCA model in 2010 were as follows: number of rooms per capita, access to piped drinking water, access to internet, use of natural gas for cooking, access to a heating and cooling system, type of house ownership, and having bathroom, refrigerator, freezer, refrigerator-freezer, color TV, LCD/LED/Plasma TV, landline, microwave, vacuum cleaner, personal computer/laptop, radio, cell phone, car, motorcycle, bicycle, and wrist watch. Consequently, five economic quintiles namely the poorest, poorer, middle, richer and the richest were constructed and used in the subsequent analyses. We tried to select these assets based on a list of 33 asset variables proposed by Tajik and Majdzadeh in 2014 to be used in surveys in Iran.^32^ Moreover, we tailored assets lists according to the level of living standards,^30^ in 2010. In fact, as households’ living standards in Iran have been changed over a 10-year period, two different lists of assets were chosen for 2000 and 2010.

Mother’s age at delivery was divided into seven age categories (≤14 years old; 15-19 years old; 20-24 years old; 25-29 years old; 30-34 years old; 35-39 years old, and ≥40 years old). Mother’s educational level was also grouped into three categories of illiterate, non-academic (including primary school/literacy movement, secondary school, high school and pre-university) and academic. Skilled pregnancy care was defined as care provided by gynecologists, general practitioners (GPs), educated midwives, family health experts, and other medical specialist. Skilled birth attendant was defined as birth attended by medical specialists, GPs, educated midwives, and other educated personnel. Modern contraceptives use included using condom, pill, ampule, Norplant, intrauterine device (IUD), tubal ligation, and vasectomy.

### Inequality Measurement

To measure socio-economic inequality in neonatal mortality, CI approach was applied.^19^ Decomposability of CI has led to its broad adoption as a reliable health inequality measure instead of other dispersion measures such as rate ratio.^33,34^ The CI is calculated as twice the (weighted) covariance of a health variable and a relative economic rank variable,^34^ as follows:

(1) $$C=\frac{2}{n\mu }\sum_{i=1}^{n}y_{i}R_{{}_{i}}-1$$

Where *y*_i_ denotes the dependent variable of interest (eg, neonatal mortality), *μ* indicates its means and *R*_i_ represents the fractional rank of each individual in terms of the index of household’s economic status. The CI value can vary from -1 to +1.^19^ Its negative values indicate that a health variable is more concentrated among the poor and vice versa.

Since, in this study, the neonatal mortality is a binary variable (whether the neonates were alive or not), a normalization of CI is required to measuring inequality appropriately. Two different approaches are introduced for this, ie, the Wagstaff,^35^ and Erreygers,^36^ normalizations. We employ the Wagstaff normalization since the percentage incidence of neonatal death in our samples are only 2% and 1.5% in DHS 2000 and MIDHS 2010, respectively, and it tends to work better for low-frequency binary outcomes,^16^ and also because it has a more emphasis on relative inequality.^37^ The normalized CI can be written as:

(2) $$C_{n}=\frac{c}{1-\mu }$$

### Decomposition of Inequality

The CI can be decomposed into its determinants to identify the contribution of each predictor variable to the measured health (eg, neonatal death) inequality. Following Wagstaff et al,^15^ we used a linear regression model linking neonatal mortality (*y*) to a set of *k* health determinants (*x*_k_):

(3) $$y_{i}=\alpha +\sum_{k}\beta_{k}x_{ki}+\varepsilon_{i}$$

Where *x*_ki_ is a set of *k* regressors variable for the *i*^th^ individual, *β*_k_ denotes the coefficient, and *ε*_i_ is an error term. Given the association of *y*_i_ and *x*_ki_ in equation 3, CI for (*y*) can be represented as:

(4) $$C=\sum_{k}(\frac{\beta_{k}{\bar{X}}_{k}}{\mu })C_{k}+\frac{GC_{\varepsilon }}{\mu }=C\hat{y}+\frac{GC_{\varepsilon }}{\mu }$$

Where is *μ* the mean of *y, x̄*_k_ is the mean of *x*_k_, *C*_k_ is the normalized CI for *x*_k_ defined precisely like C, is the elasticity of the neonatal mortality with explanatory variables, and GC_ε_ is the generalized CI *ε*_i_ for (residual component). In other words, equation 4 is composed of two components: the first is the deterministic or explained component which consists of two elements: (1) Elasticity $\frac{\beta_{k}{\bar{X}}_{k}}{\mu }$ as a unit-free measure of association that indicates the amount of change in dependent variable (neonatal mortality in this study) associated with one unit change in explanatory variable. (2) *C*_k_ is the normalized CI of K determinants, ie, the degree of inequality in each determinant across wealth quintiles. Since we applied Wagstaff normalization to the calculation of the overall CI, it was required to apply the same normalization to the calculation of the CI of the covariates, ie, the Wagstaff normalization was applied to both sides of the decomposition equation which allowed us to calculate the contributions to the normalized index. The second component is the unexplained or residual component. It is the part of the inequality that cannot be described by systematic variation in the determinants across economic groups (residual). To decompose, one needs initially to run an appropriate regression model to calculate coefficients (*β*_k_) of the explanatory variables.

Currently, three more common regression approaches to decomposition of inequality are used in the literature: ordinary least squares (OLS) model, probit model, and the generalized linear model (GLM).^38^ In this study, neonatal mortality was considered as a binary variable. As a result, OLS could not be applied as it does not fulfill the binomial distribution requirement; probit model was not a desired option too, as it is sensitive to the choice of the reference group and the results change as the reference group changes.^16^ However, following Yiengprugsawan et al GLM (with binomial family and identity link) permits the decomposition model to hold and creates valid coefficient estimates that do not vary based on the choice of reference categories.^38^ Therefore, GLM model was used for decomposition in our study.

### Decomposition of Changes in Inequality

At the final stage, following Wagstaff et al,^15^ changes in all the components of the decomposition (equation 4) and each determinant’s contribution to inequality (equation 5 and 6) in neonatal mortality from 1995-2000 to 2005-2010 were explored. Considering all changes in inequality, the simplest method would be subtracting the equation 3 in time *t* from equation 3 in time *t-1*:

(5) $$\begin{array}{l} \Delta C=\sum_{k}\beta_{kt}{\bar{x}}_{kt}/\mu_{t})\;C_{kt}-\sum_{k}\beta_{kt-1}{\bar{x}}_{kt-1}/\mu_{t-1})\;C_{kt-1}\\ +\;\Delta (GC_{\varepsilon t}/\mu_{t}) \end{array}$$

Applying Oaxaca’s method to equation 4 we obtain:

(6) $$\begin{array}{l} \Delta C=\sum_{k}\eta_{kt}(C_{kt}-C_{kt-1})+\sum_{k}C_{kt-1}(\eta_{kt}-\eta_{kt-1})\\ +\;\Delta (GC_{\varepsilon t}/\mu_{t}) \end{array}$$

And alternatively:

(7) $$\begin{array}{l} \Delta C=\sum_{k}\eta_{kt-1}(C_{kt}-C_{kt-1})+\sum_{k}C_{kt}(\eta_{kt}-\eta_{kt-1})\\ +\;\Delta (GC_{\varepsilon t}/\mu_{t}) \end{array}$$

Where in present study, *η*_Kt_ and *η*_Kt-1_ represent the elasticities of explanatory variables in terms of neonatal mortality in 2005-2010 and 1995-2000, respectively. Also, C_kt_ and C_kt-1_ denote the normalized CIs of explanatory variables in 2005-2010 and 1995-2000, respectively.

All analyses were conducted in STATA 12/SE.

## Results

### Descriptive Statistics

Table 1 shows descriptive statistics of neonatal death and its determinants in Iran in 1995-2000 and 2005-2010. The percentage of neonatal death in 1995-2000 compared to 2005-2010 declined from 2.19 to 1.51 (31% reduction). For explanatory variables, history of abortion/stillbirth decreased 10%, but use of skilled birth attendants and possession of a hygienic toilet increased around 65% and 83%, respectively. There was also an increase in urbanization rate (about 47% in 1995-2000 vis-a-vis 64% in 2005-2010). In terms of mother’s education, illiteracy rate dropped from 29% in 1995-2000 to 9% in 2005-2010, whereas academic education rate increased from 4% to 11% over that period. This table also shows means of various determinants of neonatal health that were included into the regression model as explanatory variables.

**Table 1:** Summary Statistics for Neonatal Death and its Determinants in Iran, 1995-2000 and 2005-2010

**Variable**	**1995-2000, n = 45 646**			**2005-2010, n = 10 604**		
	**Mean**	**SD**	**%**	**Mean**	**SD**	**%**
Neonatal death	0.0199	0.1399	2.19	0.0149	0.1213	1.51
Household economic status						
Poorest	0.2504	0.4000	25.00	0.2456	0.4276	25.00
Poorer	0.2354	0.4097	24.00	0.2320	0.4209	23.00
Middle	0.1946	0.3856	19.00	0.2188	0.4117	22.00
Richer	0.1822	0.4055	18.00	0.1743	0.3816	17.00
Richest	0.1374	0.3969	14.00	0.1355	0.3423	13.00
Location of residence						
Urban	0.4650	0.4976	46.50	0.6404	0.4761	64.00
Rural	0.5349	0.4976	53.50	0.3596	0.4761	36.00
Child gender						
Male	0.5174	0.4997	52.00	0.5238	0.4994	52.00
female	0.4996	0.4997	48.00	0.4762	0.4994	48.00
Mother’s educational level						
Illiterate	0.2923	0.4408	29.00	0.0924	0.2897	9.00
Non-academic	0.6679	0.4632	67.00	0.7983	0.400	80.00
Academic	0.0396	0.2134	4.00	0.1093	0.3112	11.00
Mother’s age at child birth						
˂15	0.0273	0.1599	3.00	0.0438	0.2031	4.00
15-19	0.1096	0.3111	11.00	0.0945	0.2952	9.00
20-24	0.2501	0.4344	25.00	0.2847	0.4517	28.00
25-29	0.2602	0.4398	26.00	0.2986	0.4570	30.00
30-34	0.1709	0.3785	17.00	0.1824	0.3854	18.00
35-39	0.0926	0.2870	9.00	0.0745	0.2626	7.00
≥40	0.0889	0.2810	9.00	0.0215	0.1444	2.00
Skilled prenatal care						
Use	0.4330	0.4944	43.00	0.4088	0.4914	40.00
Not use	0.5670	0.4944	57.00	0.5912	0.4914	60.00
Skilled birth attendants						
Use	0.2575	0.4306	26.00	0.4275	0.4947	43.00
Not use	0.7424	0.4306	74.00	0.5725	0.4947	57.00
Modern contraceptive						
Use	0.6395	0.4821	64.00	0.6320	0.4820	63.00
Not use	0.3605	0.4821	36.00	0.3680	0.4820	37.00
History of abortion/stillbirth						
Have	0.2037	0.4050	20.00	0.1816	0.3854	18.00
Not have	0.7963	0.4050	80.00	0.8184	0.3854	82.00
Risky birth interval						
Have	0.0246	0.1466	2.00	0.0615	0.0639	6.00
Not have	0.9754	0.1466	80.00	0.9385	0.0639	94.00
aving a hygienic toilet						
Have	0.2356	0.4518	24.00	0.4400	0.4980	44.00
Not Have	0.7644	0.4518	76.00	0.5600	0.4980	56.00

Abbreviation: SD, standard deviation.

### Concentration Indices

Table 2 shows the normalised CIs for neonatal mortality in 1995-2000 and 2005-2010. The normalised CI in 1995-2000 was -0.1490 and -0.1254 in 2005-2010, showing that burden of neonatal mortality was higher among the disadvantaged households in both years. More importantly, socio-economic inequality in neonatal mortality has declined (0.0236) over the studied period. Interpretation of positive sign of the difference between CIs is slightly more complicated than usual, ie, this positive value means that inequality in neonatal mortality has moved 0.0236 unit closer to equality line in that period. In other words, the amount of inequality has reduced by 16% between 1995-2000 and 2005-2010.

**Table 2:** Normalized Concentration Indices of Neonatal Mortality in Iran, 1995-2000 and 2005-2010

**Index**	**Index Value**		**Robust SE**		***P*** **Value**		***CI ***_Y2_ ***-CI ***_Y1_
	**Y**_1_	**Y**_2_	**Y**_1_	**Y**_2_	**Y**_1_	**Y**_2_	
Wagstaff normalized CI	-0.1490	-0.1254	0.0307	0.0451	<.001	.005	0.0236

Abbreviations: SE, standard error; CI, concentration index. Note: Y_1_ and Y_2_ denote years 1995-2000 and 2005-2010, respectively.

### Decomposition of Concentration Indices

The results of decomposition of inequality in neonatal mortality in 1995-2000 and 2005-2010 are reported in Table 3. In fact, the table shows the following: the coefficients of regressors (estimated by GLM), the CIs of regressors (*C*_k_*)*, the absolute and percentage contributions of explanatory variables as well as their changes. The CIs of explanatory variables revealed that residence in rural areas, mother’s illiteracy, delivery at lower ages (<15 and 15-19), history of abortion/stillbirth, and risky birth interval all were more concentrated among people of lower economic status in 1995-2000 and 2005-2010. In contrast, mother’s education, delivery at 20-29 years old, use of skilled prenatal care, skilled birth attendants, and owning a hygienic toilet all were more concentrated among people of higher economic status in both years.

**Table 3:** Decomposition of Inequality in Neonatal Mortality in Iran (1995-2000 and 2005-2010) and Their Changes

	**Coefficient**		**Elasticity**		***C***_k_		**Absolute Contribution to CI**		**% Contribution**		**Change**
	**Y**_1_	**Y**_2_	**Y**_1_	**Y**_2_	**Y**_1_	**Y**_2_	**Y**_1_	**Y**_2_	**Y**_1_	**Y**_2_	
Household economic status											
Poorest	0.0026	0.0026	0.0327	0.0429	-0.7988	-0.7398	-0.0261	-0.0317	18	25	-0.0056
Poorer	0.0035	0.0045	0.0414	0.0701	-0.3841	-0.2659	-0.0159	-0.0186	11	15	-0.0027
Middle	0.0015	-0.0025	0.0147	-0.0367	0.1126	0.1605	0.0017	-0.0059	-1	5	-0.0075
Richer	-0.0001	-0.0009	-0.0008	-0.0104	0.5077	0.5222	-0.0004	-0.0054	0	4	-0.0050
Richest^a^	-	-	-	-	-	-	-	-	-	-	-
Sum									28	49	-0.0209
Residence in rural	0.0002	0.0037	0.0054	0.0893	-0.2693	-0.2864	-0.0014	-0.0256	1	20	-0.0241
Child gender (male)	0.0033	0.0033	0.0858	0.1160	-0.0022	0.004	-0.0002	0.0005	0	0	0.0007
Mother’s education											
Illiterate	0.0120	0.0060	0.1763	0.0372	-0.3557	-0.4979	-0.0627	-0.0185	42	15	0.0442
Non-academic	0.0044	-0.0017	0.1477	-0.0911	0.0959	0.0168	0.0142	0.0015	-10	1	-0.0157
Academic^a^	-	-	-	-	-	-	-	-	-	-	
Sum									32	16	0.0285
Mother’s age at time of delivery											
˂15	0.0179	-0.0024	0.0246	-0.0071	-0.0859	-0.0617	-0.0021	0.0004	1	0	0.0025
15-19	0.0099	-0.0005	0.0545	-0.0032	-0.0300	-0.2052	-0.0016	0.0007	1	-1	0.0023
20-24	0.0016	0.0037	0.0201	0.0707	-0.0096	-0.0390	-0.0002	-0.0028	0	2	-0.0026
25-29	-0.0058	0.0020	-0.0758	0.0401	0.0504	0.0849	-0.0038	0.0034	3	-3	0.0072
30-34	-0.0011	0.0055	-0.0094	0.0673	0.0810	0.0709	-0.0008	0.0048	1	-4	0.0055
35-39	-0.0017	-0.0041	-0.0079	-0.0205	-0.0020	0.0650	0.0000	-0.0013	0	1	-0.0013
≥40^a^	-	-	-	-	-	-	-	-	-	-	-
Sum									6	-4	0.0137
Using skilled prenatal care	0.0060	0.0124	0.1306	0.3402	0.0036	0.0037	0.0005	0.0013	0	- 1	0.0008
Using skilled birth attendants	-0.0038	0.0146	-0.0492	0.4189	0.0043	0.0439	-0.0002	0.0184	0	-15	0.0186
Using modern contraceptive	-0.0121	-0.0132	-0.3888	-0.5599	0.0144	-0.0021	-0.0056	0.0012	4	-1	0.0068
History of abortion/stillbirth	0.0111	0.0157	0.1136	0.1914	-0.0144	-0.0240	-0.0016	-0.0460	1	4	-0.0030
History of risky birth interval	-0.0067	-0.0138	-0.0083	-0.0570	-0.0256	-0.0020	0.0002	0.0001	0	0	-0.0001
Having a hygienic toilet	0.0033	0.0026	0.0391	0.0768	0.0203	0.0036	0.0008	0.0003	-1	0	-0.0005
Total observed							-0.1055	-0.0850	71	68	0.0204
Residual							-0.0435	-0.0404	29	32	0.0032
Total							-0.1490	-0.1254	100	100	0.0236

^a^Denotes reference group.

In terms of absolute contribution, if the value of the contribution of variable K is *k* and positive (negative), then the inequality in neonatal mortality would decrease (increase) by k% if the variable were to become equally distributed across the socio-economic groups. In 1995-2000, the largest contribution to inequality in neonatal mortality was attributable to mother’s education (32%), ie, if education were equally distributed among mothers belonging to different socio-economic groups, then inequality in neonatal mortality would decline by 32%. Economic status (28%), mother’s age at delivery (6%), and using modern contraceptive (4%) followed in terms of their contribution importance. In 2005-2010, the economic status made the largest contribution to inequality in neonatal mortality (49%) – if income were equally distributed among different wealth quintiles, then inequality in neonatal mortality would decrease by 49%. Furthermore, residence in rural areas (20%), mother’s education (16%), and history of abortion/stillbirth (4%) showed a substantial contribution to observed inequality, respectively (Figure).

**Figure:**
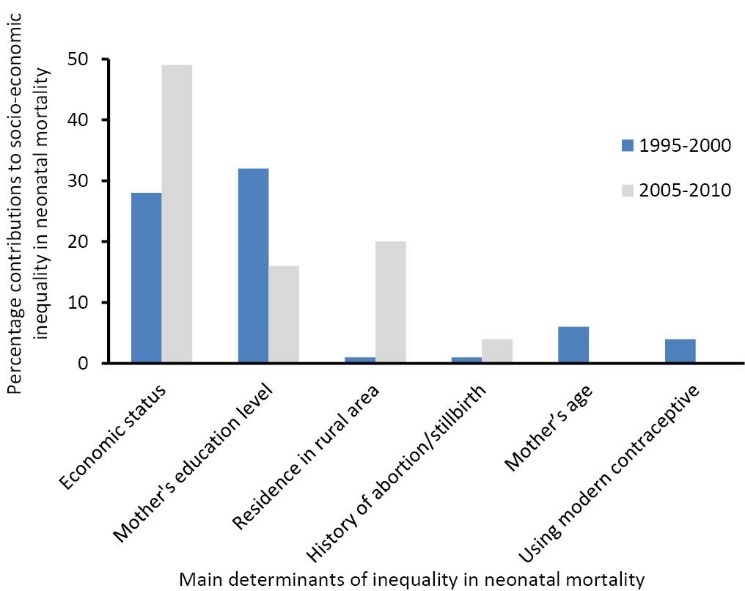
Contributions of Determinants to Socio-Economic Inequality in Neonatal Mortality in Iran (1995-2000 and 2005-2010).

The column titled change in the last column of Table 3 the empirical analogue of equation 5, reveals that the bulk of deterioration in explained inequality in neonatal mortality between 1995-2000 and 2005-2010 were due to changes in (rural area) residence status, household’s economic status, and history of abortion/stillbirth. In contrast, changes regarding mother’s education, use of skilled birth attendants, mother’s age at delivery, using the modern contraceptive, and skilled prenatal care tended to narrow observed neonatal mortality inequality in that period. Moreover, changes in risky birth interval and possession of hygienic toilet were negligible.

Table 4 also shows the residual components. The overall normalized CIs of neonatal mortality in 1995-2000 and 2005-2010 were -0.1490 and -0.1254, respectively. The explained components of the overall normalized CIs (the first term of equation 4) were -0.1055 in 1995-2000 and -0.0850 in 2005-2010. These components show that neonatal health variables entered into the current model were able to explain 71% and 68% of the measured inequalities in neonatal mortality in Iran in 1995-2000 and 2005-2010, respectively. The rest of the inequalities (29% and 32%) were residual components (the second term of equation 4) of overall normalized CIs that had values of -0.0435 and -0.0404. The residual shows the portion of neonatal mortality inequality that cannot be determined by systematic variation in the explanatory variables across socio-economic groups, consequently, it cannot be decomposed. Namely, there are other determinants that responsible for this unexplained part of inequality, but the data for those determinants were not collected.

**Table 4:** Oaxaca–Type Decomposition for Change in Inequality of Neonatal Mortality in Iran Between 1995- 2000 and 2005-2010

	**Equation 6**		**Equation 7**		**Total**	
	**∆C.η**_kt_	**∆η.C**_Kt-1_	**∆C.η**_kt–1_	**∆η.C**_Kt_	**Total**	**%**
Household economic status						
Poorest	0.0025	-0.0081	0.0019	-0.0075	-0.0056	-24
Poorer	0.0083	-0.0110	0.0049	-0.0076	-0.0027	-12
Middle	-0.0018	-0.0058	0.0007	-0.0082	-0.0075	-32
Richer	-0.0002	-0.0049	0.0000	-0.0050	-0.0050	-21
Sum	0.0089	-0.0298	0.0075	-0.0284	-0.0209	-88
Residence in rural areas	-0.0015	-0.0226	-0.0001	-0.0240	-0.0241	-102
Child gender (male)	0.0007	-0.0001	0.0005	0.0001	0.0007	3
Mother’s education						
Illiterate	-0.0053	0.0495	-0.0251	0.0692	0.0442	187
Non-academic	0.0072	-0.0229	-0.0117	-0.0040	-0.0157	-66
Sum	0.0019	0.0266	-0.0367	0.0652	0.0285	121
Mother’s age at delivery time						
˂15	-0.0002	0.0027	0.0006	0.0020	0.0025	11
15-19	0.0006	0.0017	-0.0096	0.0118	0.0023	10
20-24	-0.0021	-0.0005	-0.0006	-0.0020	-0.0026	-11
25-29	0.0014	0.0058	-0.0026	0.0098	0.0072	31
30-34	-0.0007	0.0062	0.0001	0.0054	0.0055	23
35-39	-0.0014	0.0000	-0.0005	-0.0008	-0.0013	-6
Sum	-0.0024	0.0160	-0.0126	0.0263	0.0137	58
Using skilled prenatal care	0.0000	0.0008	0.0000	0.0008	0.0008	3
Using skilled birth attendants	0.0166	0.002	-0.0019	0.0205	0.0186	79
Using modern contraceptive	0.0092	-0.0025	0.0064	0.0004	0.0068	29
History of abortion/stillbirth	-0.0018	-0.0011	-0.0011	-0.0019	-0.0030	-13
History of risky birth interval	-0.0013	0.0012	-0.0002	0.0001	- 0.0001	0
Having a hygienic toilet	-0.0013	0.0008	-0.0007	0.0001	-0.0005	-2
Total observed	0.0290	-0.0086	-0.0388	0.0593	0.0204	87
Residual					0.0032	13
Total					0.0236	100

### Oaxaca Decomposition

Table 4 illustrates Oaxaca-type decomposition results. What the last column of Table 3 fails to demonstrate is the amount of change in neonatal mortality inequality that was either due to alterations in elasticities of determinants (∆*η*) or changes in the unequal distribution of determinants (∆*C*). The second and fourth columns of Table 4 show changes in the amount of inequality in determinants; and the third and fifth columns show changes in elasticities of determinants – in correspondence to equations 6 and 7 – respectively. The total and percentage change for each determinant is indicated in the last two columns of the table. Interestingly, in variables of “residence in rural area” and “history of abortion/stillbirth,” the changes in elasticities and inequalities reinforced each other’s effect. Also, as it is shown, it was the change of elasticity rather than the unequal distribution of rural area residency that accounted for the majority of the increase in neonatal mortality inequality.

Overall, regarding the changes in all determinants of neonatal mortality, changing inequalities and changing elasticities contributed differently to the reduction in neonatal mortality inequality. Moreover, mother’s education and use of skilled birth attendants accounted for the largest contributions to the observed decrease in inequality. These variables led to 121% and 79% reduction in neonatal mortality inequality. Changes in mother’s age at delivery (25-34 years old), using the modern contraceptive, and skilled prenatal care were followed regarding their importance for the decline in neonatal mortality inequality.

The observed total inequality change (87%) refers to change in neonatal mortality inequality that was explained by variables entered into the Oaxaca decomposition model. The rest of change in inequality (13%) was a residual component of overall inequality change (0.0236).

## Discussion

In the present study, we tried to explore changes in socio-economic inequality in neonatal mortality from 1995-2000 to 2005-2010 in Iran. The main findings were as follows: there was a pro-rich inequality in neonatal mortality in both years; inequality in neonatal mortality decreased over time; the main contributors to neonatal mortality have changed over that period; improvement in mother’s education and use of skilled birth attendants were accountable for bulk of narrowing in neonatal mortality inequality in Iran.

Inequality in Iranian neonates’ mortality did favor advantaged households in 1995-2000 and 2005-2010. This finding is consistent with other studies undertaken all over the globe.^2-9^ Notably, although pro-rich inequality remains, inequality in neonatal mortality declined by 16% in Iran between 1995-2000 and 2005-2010, just like in Cameroon, Nigeria, Malawi, Mozambique, Uganda,^6^ and in Chile.^3^ In recent years, healthcare system in Iran has successfully launched some primary healthcare-based programs such as IMCI, well baby care, 1-59 months child mortality surveillance system, integrated mothers’ healthcare, and baby-friendly hospital initiative that gradually have removed geographical and financial barriers to newborn and maternal health services.^39,40^ The authors postulate that it is highly probable that such initiatives have helped in reduction of neonatal mortality inequality in Iran.

Decomposition approach showed that the largest contributors to neonatal mortality inequality have changed from 1995-2000 to 2005-2010 in Iran. In fact, inequalities in mother’s education and household’s economic status were the largest contributors to neonatal mortality inequality in 1995-2000 and 2005-2010, respectively. This finding suggests that the change in neonatal mortality inequality is most sensitive to these two important determinants. Reduction in mother’s education contribution to inequality (by 50%) over those periods can be due to comprehensive schooling policies such as free/compulsory pre-academic education and rural primary schooling and “literacy movement” in Iran. Literacy rate rose from 75.9% in 2000 to 80.07% in 2010 for female aged over 6.^24^ Moreover, the literacy rate of women aged 15-24 reached to 96.83 % in 2010. Interestingly enough, tertiary education enrolment rate (aged 15-18) among women (72.07%) have reached, approximately, to that of men (73.07%).^24^ Totally speaking, schooling policies in Iran have led to an increase in mean of schooling years,^41^ equal enrolment rate for girls and boys, and better female educational attainment in recent decades and all of these have resulted in shrinkage of educational inequality between the poor and non-poor, male and female, and rural and urban areas. Consequently, revealed reduction in the contribution of mother’s education to neonatal mortality inequality seems almost fathomable.

In contrast, the contribution of economic status to neonatal mortality inequality increased by 75% from 1995-2000 to 2005-2010. The increment in the contribution of economic status seems somehow unexpected as all Iran’s NDPs apparently targeted economic inequality by prioritizing underserved areas and low-income groups.^20^ Especially, one of the stated primary objectives of the fourth NDP (2005-2009) was to lessen the level of inequalities in health expenditures. However, such unexpected finding can be explained by following reasons: (1) the inflation rate increased from 12.1% in 2005 to 13.9% in 2010,^42^ and undoubtedly the poor were more affected; (2) the unemployment rate was 11.5% in 2005, whereas it raised to 13.5% in 2010.^43^ Unemployment, doubtlessly, has profound effects on individual income, living standards, utilization of goods and (health) services, and the level of economic equality; (3) the economic growth dropped from 5.7 to 4.7 in 2005-2010.^44^ That reduction had a negative effect on macroeconomics of the country so that business space and capacity shrank, reinforcing the widening gap between the poor and the rich.

Oaxaca decomposition revealed that changes in neonatal mortality inequality arise from the alteration in the interaction among the inequality determinants, ie, the factors with positive and negative signs offsetting the inequality. In the one hand, changes in several determinants specifically residence in rural areas, household’s economic status, and history of abortion/stillbirth pushed inequality in neonatal mortality towards deterioration. In the other hand, mother’s educational level, use of skilled birth attendants, mother’s age at delivery (25-34 years), using modern contraceptive and skilled prenatal care pushed inequality towards equality line. In overall, improvement in mother’s education and use of skilled birth attendants made the largest contributions to decline of inequality in neonatal mortality between 1995-2000 and 2005-2010. For variables of residence in rural areas and household’s economic status, changes in inequality in neonatal mortality were not so much due to shifts in the unequal distribution of these determinants, but to variations in their elasticity with neonatal mortality.

Regarding these findings, it can be suggested that improvement in mother’s education and use of skilled birth attendants are apt options to further reduction in neonatal mortality inequality. Furthermore, two main entry points for policy action on inequality in neonatal mortality are improvements in rural settings and also households’ economic status.

## Strengths and Limitations

In the present study, instead of using direct measures (eg, income, expenditure, or consumption), economic status was measured by using an indirect measure, ie, PCA. As data collection for direct measures is expensive and often somehow impractical and biased, especially in developing countries, data on household durable assets were used to create a proxy measure of economic status.^30,45^ We applied information on households’ ownership of durable goods and housing characteristics to lessen the above-raised concerns. So, comparing to other studies that used direct measures such as household’s income or expenditure to construct economic status, this study has the advantage of measuring economic status using a more accurate proxy measure. However, we must also keep in mind that using other measures of economic status may yield different estimates, especially in the magnitude and contributions to inequality. Nonetheless, our study had its caveats. First, as data were drawn from a cross-sectional study, causal interpretations should be made with caution. In fact, attribution of causality might be better explored with longitudinal or experimental data. Second, Oaxaca method cannot unravel changes in the elasticity, ie, whether changes in inequality owes more to changes in the mean of determinants or variations in the coefficients of determinants. Total differential approach (TDA),^15^ allows for changes in inequality to be decomposed into changes in means and coefficients. This type of decomposition is based on an approximation of the variation in inequality and is accurate for small changes. Combes et al,^33^ in 2011 did a sensitivity analysis using Oaxaca decomposition and TDA separately and suggested that interpretations of Oaxaca method are similar to the TDA results. The third limitation was time inconsistency, ie, death of neonates occurred years before DHS, but the households’ economic status was only measured for the year of DHS. This might have affected our results in some way, and any use or generalization of the results should bear this matter in mind. However, it seems that due to gradual nature of changes in economic status and life standards, this limitation is not of significant salience.

## Conclusion

Considering the findings, two policy approaches to tackling neonatal mortality inequality can be proposed for Iran: first, improving neonates’ health bearings in households with low socio-economic status through targeted perinatal programs. These equity-based programs such as skilled birth attendant, skilled prenatal care and modern contraceptive provision can aim to improve neonatal health in disadvantaged subgroups, including habitants of rural areas, people of lower economic status, illiterate mothers, mothers with history of abortion/stillbirth, and mothers with risky birth interval and delivery in very young or old ages.

A second approach is to address equity stratifiers including education, socio-economic position, place of residence, and gender across the whole population. Tackling inequality across the spectrum of equity stratifiers constitutes a much more comprehensive model for action on neonatal health inequality. Moreover, through this approach health policy-makers, in cooperation with other social and economic authorities, can better address equalization of economic status and improvement of maternal education to redress inequality across the whole population.

However, as these above-mentioned approaches are not mutually exclusive and are somehow complementary, adoption of both options are required in policy actions on neonatal mortality inequality.

## Acknowledgments

The present study was funded by Tehran University of Medical Sciences (TUMS), Tehran, Iran as a PhD thesis.

## Ethical issues

This study received the required ethics approval from Tehran University of Medical Sciences Research Ethics Committee, Tehran, Iran with ethical code No. 136890.

## Competing interests

Authors declare that they have no competing interests.

## Authors’ contributions

MAR was the main investigator planned the study, prepared and analyzed the data with contribution from AR and wrote the manuscript draft. AR made substantial contribution to analysis and interpretation of results and critically revised manuscript draft. AK made substantial contribution to estimates neonatal mortality, interpretation of data, and revised manuscript draft. MA had advisory role in preparation and refinement of draft. EA made important comment that helped in the interpretation of result. EKM made substantial contribution to analysis and interpretation of data and final writing of the paper.

## Authors’ affiliations

^1^Department of Health Management and Economics, School of Public Health, Tehran University of Medical Sciences, Tehran, Iran. ^2^Health Management and Economics Research Center, Isfahan University of Medical Sciences, Isfahan, Iran. ^3^Deputy of Public Health, Ministry of Health and Medical Education, Tehran, Iran. ^4^Department of Economics, Bu-Ali Sina University, Hamadan, Iran. ^5^Department of Public Health, Qom University of Medical Sciences, Qom, Iran. ^6^Centre for System Studies (CSS), Hull University Business School (HUBS), Hull York Medical School (HYMS), University of Hull, Hull, UK.

## Key messages

**Implications for policy makers**

- Socio-economic inequality disfavoring the poor exists in neonatal mortality in Iran.
- The main contributors to inequality in neonatal mortality changed over time from mother’s educational level to Socio-economic status between 1995-2000 and 2005-2010.
- Inequality in neonatal mortality has tended to decline over time, mainly, due to improvement of mother’s education and use of skilled birth attendants.

**Implications for public**

In recent years, health authorities have given more attention to tackling inequalities in health outcomes such as neonatal mortality rate (NMR). Besides achieving the desired average level in NMR, correction of unequal distribution of neonates’ death across society is of high priority. Measuring inequality in neonatal mortality, determining its contributors, and exploring inequality changes over time and across different socio-economic groups provide valuable evidence to act on neonatal mortality inequality. Therefore, we applied concentration index (CI) approach, as the most common measure of health inequalities, and decomposed it into its determinants to reveal the changes in each determinant’s contribution to inequality from 1995-2000 to 2005-2010. This study showed that the pro-rich inequality in neonatal mortality declined over time, and main contributors to inequality changed in Iran. We suggest that improvement in households’ economic status and maternal education can be two policy entry points to narrow neonatal mortality inequality in Iran.
